# Different associations of stay-at-home exposure with changes in body mass index and cardiometabolic factors depending on occupational physical activity: a longitudinal quasi-experimental design

**DOI:** 10.1093/joccuh/uiaf069

**Published:** 2025-11-28

**Authors:** Daijiro Kabata, Noriaki Kakiuchi, Takashi Marui, Naoko Ikeda, Mutsuko Kawai, Aki Kaimori, Noriko Saeki, Katsufumi Kajimoto, Riho Tanaka, Ayumi Zeniya, Fumi Yamanouchi, Saori Matsumiya, Yukihiro Koretsune

**Affiliations:** Center for Mathematical and Data Science, Kobe University, Kobe, Japan; Safety & Health Care Department, Daihatsu Health Care Center, Daihatsu Motor Co, Ltd, Osaka, Japan; Safety & Health Care Department, Daihatsu Health Care Center, Daihatsu Motor Co, Ltd, Osaka, Japan; Safety & Health Care Department, Daihatsu Health Care Center, Daihatsu Motor Co, Ltd, Osaka, Japan; Safety & Health Care Department, Daihatsu Health Care Center, Daihatsu Motor Co, Ltd, Osaka, Japan; Safety & Health Care Department, Daihatsu Health Care Center, Daihatsu Motor Co, Ltd, Osaka, Japan; Safety & Health Care Department, Daihatsu Health Care Center, Daihatsu Motor Co, Ltd, Osaka, Japan; Safety & Health Care Department, Daihatsu Health Care Center, Daihatsu Motor Co, Ltd, Osaka, Japan; Safety & Health Care Department, Daihatsu Health Care Center, Daihatsu Motor Co, Ltd, Osaka, Japan; Safety & Health Care Department, Daihatsu Health Care Center, Daihatsu Motor Co, Ltd, Osaka, Japan; Safety & Health Care Department, Daihatsu Health Care Center, Daihatsu Motor Co, Ltd, Osaka, Japan; Safety & Health Care Department, Daihatsu Health Care Center, Daihatsu Motor Co, Ltd, Osaka, Japan; Safety & Health Care Department, Daihatsu Health Care Center, Daihatsu Motor Co, Ltd, Osaka, Japan

**Keywords:** occupational physical activity, stay-at-home, body mass index, cardiometabolic risk, interrupted time-series analysis

## Abstract

**Objectives:**

To quantify the short-term impact of an unexpected stay-at-home exposure, caused by a shipment suspension, on body mass index (BMI) and cardiometabolic markers among employees with different levels of occupational physical activity.

**Methods:**

Health-check records from 8307 workers at a large Japanese automobile manufacturer were linked to company attendance data covering a shipment suspension (January to April 2024). An interrupted time-series assessed BMI trajectories before, during, and after the halt. Among 614 employees who underwent an additional examination in April 2024, mixed-effects models related the duration of stay-at-home to changes in BMI and blood pressure within low-, medium-, and high-intensity job categories.

**Results:**

Compared with pre-halt trends, medium-intensity and high-intensity workers showed significant level rises in BMI (0.96 kg/m^2^; 95% CI, 0.56-1.36; and 0.64 kg/m^2^; 95% CI, 0.24-1.04, respectively) at the onset of the suspension. Mixed-effects analyses showed a positive dose–response between the duration of stay-at-home and BMI gain in high-intensity jobs (0.47 kg/m^2^ per 20% absent days; 95% CI, 0.37-0.58). Per 20% of scheduled workdays absent, systolic blood pressure was higher in the medium- and high-intensity groups. No significant effects were observed among sedentary workers.

**Conclusions:**

Employees whose daily energy expenditure relies on job-related physical activity are especially susceptible to weight gain and blood pressure elevations during forced work interruptions. Business continuity plans should embed tailored countermeasures—such as structured exercise programs and phased returns to on-site duties—to safeguard metabolic health during future operational disruptions.

## Teaser

Key points:

- **What is already known on this topic**: Pandemic-era studies have suggested that stay-at-home orders reduce physical activity and promote weight gain, but evidence is scarce for manual-labor employees whose daily energy expenditure depends on their jobs; previous analyses were confounded by infection-control policies.
- **What this study adds**: Exploiting an unexpected 4-month shipment suspension at a Japanese automobile plant, this interrupted time-series and mixed-effects analysis of 8307 workers showed that BMI and systolic blood pressure rose most sharply among high-intensity (physically demanding) occupations, with a dose–response relationship between days absent and cardiometabolic deterioration, whereas sedentary workers were largely unaffected.
- **How this study might affect research, practice, or policy**: The findings indicate that business continuity and occupational health plans should embed targeted countermeasures—structured at-home exercise, phased on-site returns, rotating shifts, and stress-management resources—to preserve metabolic health during future work stoppages. We provide a quasi-experimental framework for evaluating such interventions.

## 1. Introduction

Sudden workplace closures, such as unexpected plant shutdowns or shipment suspensions, can dramatically alter employees’ daily work routines and lifestyles. Prior literature from the COVID-19 era highlighted that stay-at-home orders and telework restrictions may lead to reduced physical activity, changes in dietary patterns, and increased psychological stress, ultimately posing risks for weight gain and cardiometabolic deterioration.^1,2^ However, most pandemic-related studies have focused on employees in occupations amenable to remote work, leaving limited insight into outcomes for workers in positions that require substantial physical labor. Workers engaged in high-intensity or heavy manual labor rely heavily on occupational physical activity to maintain daily energy expenditure; thus, abrupt periods of forced inactivity may disproportionately affect this subgroup.^3^

Moreover, the complex interplay of public health policies, varying infection rates, and other external factors during the COVID-19 pandemic makes it difficult to isolate the pure effect of forced inactivity on health indicators. By contrast, business-driven shutdowns or shipment suspensions often occur independently of health policies or broader societal shifts,^4^ thereby offering a setting in which quasi-experimental approaches, such as analysis based on an interrupted time-series (ITS) design, can more robustly estimate the impact of abrupt changes in occupational activity.^5,6^ Specifically, these scenarios allow researchers to evaluate short-term “stay-at-home” exposures without the major confounding influences that accompany large-scale policy or environmental shifts.^7^

In this study, we exploited a unique situation in which a major Japanese automobile manufacturer abruptly suspended shipments from January to April 2024. For clarity, the factory shutdown analyzed here was unrelated to COVID-19 or public health mandates. The resulting forced work stoppage required many employees—across a wide range of job categories, including those with high physical demands—to stay at home for extended periods. Because annual health examinations at this company are conducted according to birth month, the schedule of medical checkups remained essentially unchanged before, during, and after the shutdown. This operational characteristic facilitated an ITS design by enabling comparison of employees’ health parameters across distinct time points surrounding the forced disruption.

The primary objective of this investigation was to assess whether the sudden stay-at-home exposure was associated with short-term changes in health indices, including body mass index (BMI), blood pressure, and metabolic biomarkers. A secondary goal was to evaluate whether workers engaged in occupations characterized by high-intensity physical activity exhibited a greater risk of adverse health outcomes compared with those in sedentary or low-intensity roles.^5,6^ These findings may inform how employers and occupational health stakeholders should address future instances of forced inactivity—arising from business decisions, natural disasters, or other disruptions—by tailoring interventions to an individual’s baseline level of occupational activity.

## 2. Materials and methods

### 2.1. Study design

This retrospective cohort study analyzed longitudinal data collected from employees of a major Japanese automobile manufacturer, between January 2023 and November 2024. All data regarding health information were obtained from mandatory annual health checkups, which are a legal requirement for most full-time employees in Japan. Each employee generally undergoes a health checkup in the month of his or her birthday, ensuring that most months of the year involve some health examination activity. During the study timeframe, no major policy changes (such as new health campaigns or government-mandated interventions) were introduced in the company, apart from the suspension triggered by shipment issues in January 2024.

### 2.2. Ethics statement

This study involving human participants was reviewed and approved by the Institutional Review Board II at the National Hospital Organization, Osaka National Hospital, Japan (approval No. 23115). All methods were carried out in accordance with relevant guidelines and regulations, including the principles of the Declaration of Helsinki. Employees were informed of their right to opt out at any stage, and any employee data from individuals who opted out were excluded from the analysis.

### 2.3. Study population

The study population consisted of individuals who received a health checkup in 2023 and met the following criteria: no history of stroke, cardiovascular disease, or kidney disease; no pharmacological treatments for hypertension, diabetes, or dyslipidemia; no receipt of specific health guidance (a program in Japan designed for individuals considered at elevated risk for metabolic syndrome); no sick leave during the study period; and no misrecorded workdays. In total, 8307 employees satisfied the eligibility requirements, and their data were incorporated into the initial analyses focusing on BMI trends over time.

### 2.4. Outcome measurements

The primary outcome of interest was BMI (kg/m^2^). Secondary outcomes included systolic blood pressure (SBP, mmHg), diastolic blood pressure (DBP, mmHg), hemoglobin A1c (HbA1c, %), triglycerides (mg/dL), high-density lipoprotein (HDL) cholesterol (mg/dL), low-density lipoprotein (LDL) cholesterol (mg/dL), and uric acid (mg/dL). These variables were measured during the annual health checkups as standard practice.

### 2.5. Statistical analysis

The demographic and clinical characteristics of the study population, assessed in each period, are summarized in Table 1, with median and interquartile range (IQR) for continuous variables, and proportion and count of each category for categorical variables.

**Table 1:** Demographic and clinical characteristics by period.^a^

	**Period 1** **(Jan-Dec 2023; 12 monthly observation points)**				**Period 2** **(Jan-Apr 2024; 4 monthly observation points)**				**Period 3** **(May-Nov 2024; 7 monthly observation points)**			
	**Overall**	**[1] Low**	**[2] Medium**	**[3] High**	**Overall**	**[1] Low**	**[2] Medium**	**[3] High**	**Overall**	**[1] Low**	**[2] Medium**	**[3] High**
	***n* = 8307**	***n* = 3360**	***n* = 1534**	***n* = 3413**	***n* = 2454**	***n* = 989**	***n* = 451**	***n* = 1014**	***n* = 4218**	***n* = 1816**	***n* = 800**	***n* = 1602**
Age, y	38.02 (31.05, 46.99)	39.96 (31.98, 49.99)	39.02 (33.96, 47.95)	36.03 (28.01, 43.99)	39.91 (33.96, 48.03)	41.00 (33.08, 52.01)	40.98 (34.98, 48.10)	37.98 (33.00, 44.08)	40.01 (33.97, 48.07)	41.10 (34.02, 50.95)	40.01 (34.45, 48.08)	38.02 (33.00, 45.06)
(Missing)	0% (0)	0% (0)	0% (0)	0% (0)	0% (0)	0% (0)	0% (0)	0% (0)	0% (0)	0% (0)	0% (0)	0% (0)
Sex (male)	91% (7520)	83% (2772)	94% (1448)	97% (3300)	91% (2234)	83% (816)	95% (428)	98% (990)	90% (3815)	83% (1514)	94% (753)	97% (1548)
(Missing)	0% (0)	0% (0)	0% (0)	0% (0)	0% (0)	0% (0)	0% (0)	0% (0)	0% (0)	0% (0)	0% (0)	0% (0)
BMI, kg/m^2^	22.03 (20.24, 24.03)	22.11 (20.29, 24.01)	22.37 (20.67, 24.37)	21.73 (19.99, 23.87)	22.40 (20.60, 24.51)	22.09 (20.46, 24.14)	22.89 (20.90, 24.82)	22.49 (20.62, 24.64)	22.32 (20.48, 24.41)	22.22 (20.40, 24.24)	22.57 (20.90, 24.50)	22.31 (20.35, 24.55)
(Missing)	0.1% (12)	0.1% (4)	<0.1% (1)	0.2% (7)	0.1% (3)	0% (0)	0.2% (1)	0.2% (2)	0.1% (6)	<0.1% (1)	0.3% (2)	0.2% (3)
Alcohol												
[1] Nondrinker	27% (2249)	23% (775)	23% (350)	33% (1124)	25% (618)	21% (205)	23% (102)	31% (311)	21% (902)	18% (327)	16% (131)	28% (444)
[2] Occasional	23% (1938)	24% (808)	22% (330)	24% (800)	26% (650)	25% (251)	28% (125)	27% (274)	31% (1321)	33% (600)	31% (246)	30% (475)
[3] <3 d/wk	22% (1857)	27% (904)	23% (345)	18% (608)	22% (530)	27% (266)	19% (86)	18% (178)	16% (671)	19% (337)	16% (132)	13% (202)
[4] ≥3 d/wk	13% (1097)	14% (460)	16% (241)	12% (396)	14% (337)	15% (150)	15% (67)	12% (120)	17% (723)	17% (318)	19% (150)	16% (255)
[5] Every day	14% (1150)	12% (412)	17% (267)	14% (471)	13% (319)	12% (117)	16% (71)	13% (131)	14% (603)	13% (236)	18% (142)	14% (225)
(Missing)	0.2% (16)	<0.1% (1)	<0.1% (1)	0.4% (14)	0% (0)	0% (0)	0% (0)	0% (0)	0.8% (34)	0.9% (16)	0.9% (7)	0.7% (11)
Smoking												
[1] Never	53% (4380)	65% (2180)	46% (704)	44% (1496)	53% (1305)	65% (644)	46% (206)	45% (455)	62% (2617)	72% (1316)	57% (461)	52% (840)
[2] Past	18% (1475)	18% (617)	20% (311)	16% (547)	17% (420)	17% (171)	21% (93)	15% (156)	11% (448)	12% (213)	13% (107)	7.9% (128)
[3] Current	29% (2446)	17% (562)	34% (516)	40% (1368)	30% (729)	18% (174)	34% (152)	40% (403)	28% (1183)	16% (302)	30% (238)	40% (643)
(Missing)	<0.1% (6)	<0.1% (1)	0.2% (3)	<0.1% (2)	0% (0)	0% (0)	0% (0)	0% (0)	0.1% (6)	0.2% (3)	0.2% (2)	<0.1% (1)
Exercise (yes)	31% (2568)	31% (1030)	29% (443)	32% (1095)	29% (701)	29% (291)	28% (126)	28% (284)	33% (1402)	33% (592)	31% (247)	35% (563)
(Missing)	<0.1% (8)	0.1% (4)	0.2% (3)	<0.1% (1)	<0.1% (1)	0% (0)	0% (0)	<0.1% (1)	0% (0)	0% (0)	0% (0)	0% (0)
Commute												
Bicycle	11% (906)	14% (482)	12% (186)	7.0% (238)	9.8% (240)	14% (134)	8.0% (36)	6.9% (70)	11% (446)	14% (256)	10% (82)	6.7% (108)
Car/motor bike	53% (4383)	41% (1366)	58% (891)	62% (2126)	58% (1425)	43% (421)	65% (293)	70% (711)	56% (2346)	43% (788)	58% (465)	68% (1093)
Train/Bus	21% (1737)	30% (1006)	17% (263)	14% (468)	18% (451)	29% (285)	17% (77)	8.8% (89)	19% (815)	29% (521)	19% (148)	9.1% (146)
Walking	15% (1274)	15% (500)	13% (194)	17% (580)	14% (338)	15% (149)	10% (45)	14% (144)	14% (609)	14% (249)	13% (105)	16% (255)
(Missing)	<0.1% (7)	0.2% (6)	0% (0)	<0.1% (1)	0% (0)	0% (0)	0% (0)	0% (0)	<0.1% (2)	0.1% (2)	0% (0)	0% (0)
Location												
Osaka 1	46% (3817)	79% (2669)	49% (745)	12% (403)	45% (1096)	77% (759)	45% (205)	13% (132)	47% (1974)	78% (1415)	47% (379)	11% (180)
Osaka 2	5.4% (448)	9.3% (311)	3.2% (49)	2.6% (88)	5.0% (122)	8.5% (84)	2.9% (13)	2.5% (25)	5.8% (244)	9.3% (169)	3.1% (25)	3.1% (50)
Kyoto	9.8% (811)	1.5% (52)	8.7% (133)	18% (626)	9.7% (239)	2.5% (25)	9.8% (44)	17% (170)	11% (445)	2.4% (43)	9.4% (75)	20% (327)
Shiga 1	20% (1643)	5.0% (167)	22% (342)	33% (1134)	22% (536)	5.7% (56)	23% (105)	37% (375)	18% (780)	5.2% (94)	19% (155)	33% (531)
Shiga 2	19% (1588)	4.8% (161)	17% (265)	34% (1162)	19% (461)	6.6% (65)	19% (84)	31% (312)	18% (775)	5.2% (95)	21% (166)	32% (514)
(Missing)	0% (0)	0% (0)	0% (0)	0% (0)	0% (0)	0% (0)	0% (0)	0% (0)	0% (0)	0% (0)	0% (0)	0% (0)
Cardiometabolic factors												
HbA1c, %	5.50 (5.30, 5.70)	5.50 (5.30, 5.70)	5.50 (5.30, 5.70)	5.60 (5.40, 5.70)	5.60 (5.40, 5.80)	5.50 (5.30, 5.70)	5.60 (5.40, 5.80)	5.60 (5.40, 5.80)	5.50 (5.30, 5.70)	5.50 (5.30, 5.60)	5.50 (5.30, 5.70)	5.50 (5.30, 5.70)
(Missing)	30% (2458)	25% (838)	25% (384)	36% (1236)	25% (614)	22% (222)	21% (95)	29% (297)	23% (977)	22% (393)	21% (167)	26% (417)
Triglycerides, mg/dL	74.00 (53.00, 109.00)	79.00 (57.00, 113.00)	79.00 (56.00, 114.00)	67.00 (47.00, 100.00)	82.00 (59.00, 120.00)	77.00 (58.50, 114.50)	85.00 (62.00, 126.00)	85.00 (58.00, 123.00)	79.00 (57.00, 118.00)	83.00 (61.00, 124.00)	84.00 (59.00, 123.00)	72.00 (51.00, 108.00)

(Continued)

**Table 1:** Continued

	**Period 1** **(Jan-Dec 2023; 12 monthly observation points)**				**Period 2** **(Jan-Apr 2024; 4 monthly observation points)**				**Period 3** **(May-Nov 2024; 7 monthly observation points)**			
	**Overall**	**[1] Low**	**[2] Medium**	**[3] High**	**Overall**	**[1] Low**	**[2] Medium**	**[3] High**	**Overall**	**[1] Low**	**[2] Medium**	**[3] High**
	***n* = 8307**	***n* = 3360**	***n* = 1534**	***n* = 3413**	***n* = 2454**	***n* = 989**	***n* = 451**	***n* = 1014**	***n* = 4218**	***n* = 1816**	***n* = 800**	***n* = 1602**
(Missing)	30% (2451)	25% (831)	25% (383)	36% (1237)	25% (613)	22% (221)	21% (95)	29% (297)	23% (977)	22% (393)	21% (167)	26% (417)
HDL cholesterol, mg/dL	60.00 (51.00, 71.00)	59.00 (50.00, 70.00)	58.00 (50.00, 68.00)	62.00 (52.00, 72.00)	61.00 (51.00, 71.00)	62.00 (52.00, 73.00)	59.00 (50.00, 69.00)	61.00 (52.00, 71.00)	60.00 (51.00, 70.00)	60.00 (51.00, 71.00)	58.00 (50.00, 69.00)	60.00 (51.00, 70.00)
(Missing)	30% (2451)	25% (831)	25% (383)	36% (1237)	25% (613)	22% (221)	21% (95)	29% (297)	23% (977)	22% (393)	21% (167)	26% (417)
LDL cholesterol, mg/dL	117.00 (98.00, 136.00)	119.00 (101.00, 139.00)	117.00 (100.00, 137.00)	113.00 (93.00, 133.00)	126.00 (106.00, 146.00)	126.00 (107.50, 145.00)	129.00 (108.00, 149.00)	125.00 (104.00, 146.00)	119.00 (99.00, 139.00)	121.00 (103.00, 140.00)	120.00 (100.00, 139.00)	115.00 (94.00, 137.00)
(Missing)	30% (2451)	25% (831)	25% (383)	36% (1237)	25% (613)	22% (221)	21% (95)	29% (297)	23% (977)	22% (393)	21% (167)	26% (417)
SBP, mmHg	116.00 (108.00, 124.00)	116.00 (107.00, 125.00)	118.00 (109.00, 126.00)	115.00 (107.00, 123.00)	118.00 (109.00, 126.00)	116.00 (107.00, 125.00)	120.00 (111.00, 128.00)	118.00 (110.00, 126.00)	116.00 (107.00, 126.00)	115.00 (106.00, 125.00)	118.00 (109.00, 127.00)	116.00 (107.00, 126.00)
(Missing)	0% (0)	0% (0)	0% (0)	0% (0)	0% (0)	0% (0)	0% (0)	0% (0)	0% (0)	0% (0)	0% (0)	0% (0)
DBP, mmHg	74.00 (67.00, 81.00)	75.00 (68.00, 82.00)	76.00 (69.00, 82.00)	72.00 (65.00, 79.00)	75.00 (68.00, 82.00)	74.00 (67.00, 82.00)	77.00 (69.00, 83.00)	74.00 (67.00, 81.00)	73.00 (66.00, 80.00)	72.00 (65.00, 80.00)	74.00 (67.00, 82.00)	72.00 (65.00, 80.00)
(Missing)	0% (0)	0% (0)	0% (0)	0% (0)	0% (0)	0% (0)	0% (0)	0% (0)	0% (0)	0% (0)	0% (0)	0% (0)
Uric acid, mg/dL	6.00 (5.10, 6.80)	5.90 (5.00, 6.70)	6.00 (5.20, 6.80)	6.00 (5.20, 6.90)	5.90 (5.10, 6.70)	5.90 (4.90, 6.60)	5.95 (5.20, 6.80)	5.90 (5.10, 6.80)	6.00 (5.20, 6.90)	5.90 (5.00, 6.80)	6.10 (5.40, 7.00)	6.10 (5.30, 6.90)
(Missing)	30% (2453)	25% (831)	25% (383)	36% (1239)	25% (613)	22% (221)	21% (95)	29% (297)	23% (977)	22% (393)	21% (167)	26% (417)

Abbreviations: BMI, body mass index; DBP, diastolic blood pressure; HbA1c, hemoglobin A1c; HDL, high-density lipoprotein; LDL, low-density lipoprotein; SBP, systolic blood pressure.

^a^Values are median (interquartile range; IQR) for continuous variables, and proportion (count) for categorical variables.

**Figure 1:**
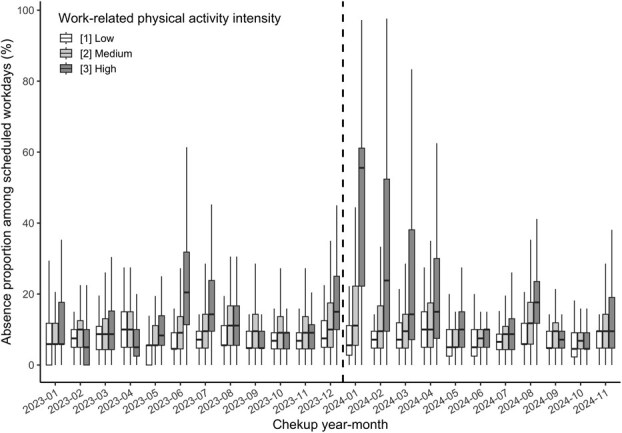
Distributions of absence proportion among scheduled working days. Box plots illustrating the distribution of absence proportion among scheduled working days. Each box plot displays the 25th, 50th, and 75th percentiles as the bottom, middle, and top lines of the box, respectively. The whiskers extend to the upper limit of the third quartile plus 1.5 times the interquartile range, and to the lower limit of the first quartile minus 1.5 times the interquartile range.

To visualize the population that did not attend the workplace during study periods, we illustrate a monthly box plot of absence proportions (Figure 1), which was computed for each employee as the proportion of missed workdays relative to the total scheduled workdays per month. This metric included both short-term absences (eg, single-day nonattendance) and prolonged leaves if they occurred within the relevant month. For the majority of employees, absences were recorded electronically through the company’s time-management system. We separately show the trend of the absence proportion for each employee group with different work-related physical activity levels. Employees whose work-related physical activities were predominantly sedentary, such as administrative or desk-bound tasks, were labeled as low-intensity. Those who performed a mixture of sedentary and physically active tasks, such as roles involving both computer work and occasional machine operation, were classified as medium-intensity. Individuals engaged primarily in physically demanding tasks on the factory floor, including assembly, manual loading, or other labor-intensive processes, were categorized as high-intensity. The level of work-related physical activity was assessed via the questionnaire administered during the annual health checkup.

To examine changes in monthly median BMI around the start of the shipment suspension in January 2024, we employed an ITS design, which is a powerful quasi-experimental design for evaluating the impact of abrupt policy or environmental changes.^5,6^ In the ITS analysis, the study period was divided into 3 segments: Period 1 (January-December 2023, pre-suspension); Period 2 (January-April 2024, during suspension); and Period 3 (May-November 2024, post-suspension). Using the segmented regression model, we estimated both the immediate shift in BMI after starting the shipment suspension (a level change at the start of Period 2) and a monthly gradient of BMI (a slope) for each period. The change in the slopes over a period can be regarded as led by the exposure in a previous period under the assumption that there are no factors modified during the study period. Because it was hypothesized that the effect of the stay-at-home exposure on BMI caused by the shipment suspension would be more pronounced for employees in physically demanding roles, the ITS models were stratified by the intensity level of work-related physical activity (low, medium, and high).

Furthermore, to assess the association of the absence proportion during the shipment suspension (from January to April 2024) on BMI and other outcomes among individuals who underwent a health checkup in April 2024, including 614 subjects, we used mixed-effects models with a random intercept based on facilities of health checkups. This model considers the outcome measure changes from the April 2023 to the April 2024 health examination as the dependent variables. To account for potential variation in the impact of absence proportion based on the physical activity characteristics associated with work-related activity, a cross-product term between the average absence proportion and the physical activity characteristics of job tasks was included. We present both unadjusted and adjusted descriptive analyses of participants’ characteristics. The adjusted models consider age, sex, drinking status (categorized as none, occasional, 1-2 days per week, 3-6 days per week, and every day), smoking status (categorized as never, past, and current), exercise habits (defined as habitual exercise at least twice per week for over a year), commute method (categorized as walking, cycling, public transportation, and car/motorbike), and health metrics measured at the 2023 health examination: HbA1c, triglycerides, HDL cholesterol, LDL cholesterol, SBP, DBP, and uric acid levels. All missing values were imputed through a multiple imputation approach based on the predicted-mean-matching method with 5 reputations.^8,9^

All hypothesis tests used a 2-sided 5% significance level, and all analyses were performed using R version 4.4.1 (https://cran.r-project.org/). We used mainly the “rms” package for the estimations and “ggplot2” for the figure depictions.

## 3. Results

### 3.1. Descriptive analysis

Table 1 shows the demographic and clinical characteristics by the observation periods. A total of 8307 participants (91% male) were included within 2023. By occupational physical activity level, 40% (*n* = 3360) were classified as low intensity, 18% (*n* = 1534) as medium intensity, and 41% (*n* = 3413) as high intensity. Overall, the median (IQR) age was 38.02 (31.05, 46.99) years, with the low-, medium-, and high-intensity groups showing median ages of 39.96 (31.98, 49.99), 39.02 (33.96, 47.95), and 36.03 (28.01, 43.99) years, respectively. Regarding alcohol consumption, 27% of participants were nondrinkers overall, but this proportion reached 33% in the high-intensity group; 14% of participants reported drinking alcohol daily. Smoking habits differed by group, with 65% never-smokers in the low-intensity group and 40% current smokers in the high-intensity group (29% overall). Regular exercise (≥2 times/week for ≥1 year) was reported by 31% of participants, and private vehicle commuting was most common (53%), especially in the high-intensity group (62%). Laboratory measures, including HbA1c, triglycerides, HDL cholesterol, and LDL cholesterol, were missing for approximately 30% of participants. Among the available measurements, median SBP was 116 mmHg (IQR: 108, 124) overall and varied little across groups. Median DBP was 74 mmHg (IQR: 67, 81) overall, slightly lower in the high-intensity group compared with those in low- or medium-intensity occupations. The inclusion of individuals in the analysis population was determined based on the 2023 health checkup; however, those who partially left the cohort due to retirement thereafter were excluded from the analysis population. Therefore, we show the distributions of characteristics assessed in Period 2 (January-April 2024; 4 monthly observation points) and Period 3 (May-November 2024; 7 monthly observation points). Across all work-related physical intensity groups, there were no substantial changes in covariates associated with the reduction in the number of participants in all periods, and thus we considered that there were no changes in group characteristics from 2023 other than those due to the passage of time. Moreover, to assess differences in participant characteristics across observation months—that is, potential seasonal influences—Table S1 presents baseline characteristics for each calendar month in 2023. Although some month-to-month fluctuations were observed, no consistent or systematic month-specific trends emerged for most factors, indicating that any seasonal or month-related variation in participant characteristics was limited.

Figure 1 and Table S2 present the distributions of absence proportions across the study period. In 2023, absence proportions were mostly 5%-10% in the low- and medium-intensity groups, whereas the high-intensity group showed several months above 10% (eg, 20% in June)—still far below the January-April 2024 surge. However, beginning January 2024, a notable shift was observed, especially in the high-intensity group, where the absence proportion surged dramatically to 56% (IQR: 22%-61%) in January, substantially higher than the preceding months. This sharp increase persisted to some extent through April 2024, with elevated absence proportions in February (24%; IQR: 10%-52%) and March (14%; IQR: 7%-38%). The medium-intensity group also experienced a moderate elevation during January 2024 (11%; IQR: 6%-22%), although the shift was less pronounced compared with the high-intensity group. In contrast, absence proportions in the low-intensity group remained relatively stable without notable changes during this period.

### 3.2. Change in BMI after the shipment suspension

**Figure 2:**
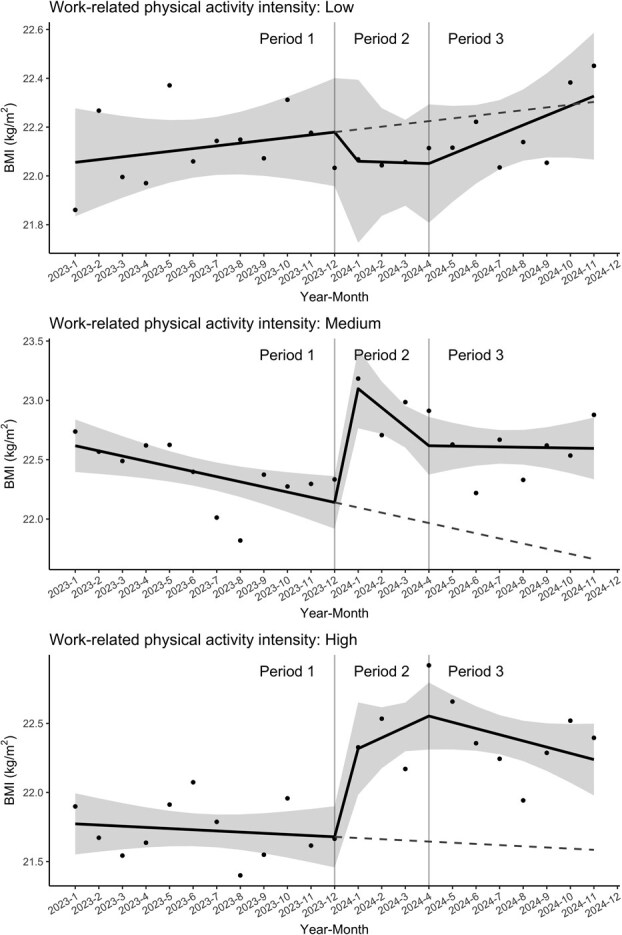
BMI trends estimated by the segmented linear regression models. The solid regression lines are estimated with the segmented linear regression model. The shaded areas are 95% CIs. The dashed lines represent the counterfactual slopes that would have continued from period 1 if no suspension had occurred.

Figure 2 and Table 2 display estimates derived from segmented regression models based on the ITS design. These estimates suggest differences in BMI trends following the start of the suspension period. Specifically, employees predominantly engaged in sedentary work (low intensity) exhibited minor changes in both the BMI trend before and after the suspension, as well as in the immediate level change following the suspension. The BMI trend in this group remained relatively stable across all periods. For employees performing mixed sedentary and physical work (medium intensity), a significant level change was observed at the onset of Period 2 (0.96; 95% CI: 0.56, 1.36; *P* < .001). However, trend changes in slopes at the onset of Period 2 and subsequently in Period 3 were not statistically significant. Employees primarily engaged in physical work (high intensity) showed a significant level change at the onset of Period 2 (0.64; 95% CI: 0.24, 1.04; *P* = .002). Nevertheless, the trend changes from Period 1 to Period 2 and to Period 3 were not statistically significant.

**Table 2:** The results of segmented linear regression models in ITS design.

**Work-related physical activity intensity**	**Period** ^**a**^	**BMI slope per month [95% CI] (*P* value)**	**Slope change from Period 1 [95% CI] (*P* value)**
**[1] Low**	Period 1	0.01 [−0.02 to 0.05] (*P* = .518)	
	Period 2	0.00 [−0.15 to 0.15] (*P* = .968)	−0.01 [−0.17 to 0.14] (*P* = .855)
	Period 3	0.04 [−0.02 to 0.10] (*P* = .193)	0.03 [−0.04 to 0.10] (*P* = .420)
**[2] Medium**	Period 1	−0.04 [−0.08 to −0.01] (*P* = .013)	
	Period 2	−0.16 [−0.31 to −0.01] (*P* = .037)	−0.12 [−0.27 to 0.04] (*P* = .138)
	Period 3	0.00 [−0.06 to 0.06] (*P* = .916)	0.04 [−0.03 to 0.11] (*P* = .250)
**[3] High**	Period 1	−0.01 [−0.04 to 0.03] (*P* = .623)	
	Period 2	0.08 [−0.07 to 0.23] (*P* = .306)	0.09 [−0.07 to 0.24] (*P* = .269)
	Period 3	−0.04 [−0.10 to 0.01] (*P* = .138)	−0.04 [−0.10 to 0.03] (*P* = .298)
**Work-related physical activity intensity**	**Timepoint**	**Predicted BMI level [95% CI]**	**Level change from the end of Period 1 [95% CI] (*P*-value)**
**[1] Low**	End of Period 1	22.18 [21.96 to 22.40]	
	Start of Period 2	22.06 [21.73 to 22.39]	−0.12 [−0.52 to 0.28] (*P* =0.559)
**[2] Medium**	End of Period 1	22.14 [21.92 to 22.36]	
	Start of Period 2	23.10 [22.76 to 23.43]	0.96 [0.56 to 1.36] (*P* < .001)
**[3] High**	End of Period 1	21.68 [21.46 to 21.90]	
	Start of Period 2	22.32 [21.98 to 22.65]	0.64 [0.24 to 1.04] (*P* = .002)

Abbreviations: BMI, body mass index; ITS, interrupted time-series.

^a^Period 1 covers the period from January to December 2023, Period 2 from January to April 2024, and Period 3 from May to November 2024. Accordingly, the estimated BMI level at the end of Period 1 refers to the estimate for December 2023, and the start of Period 2 refers to the estimate for January 2024. The difference between these two estimates is calculated as the level change from Period 1 to Period 2.

### 3.3. Association between the absence proportion and health outcomes

The mixed-effects models assessed the relationship of average absence proportion during the shipment suspension (January-April 2024) and the annual change in BMI and various clinical outcomes among 614 subjects.

**Figure 3:**
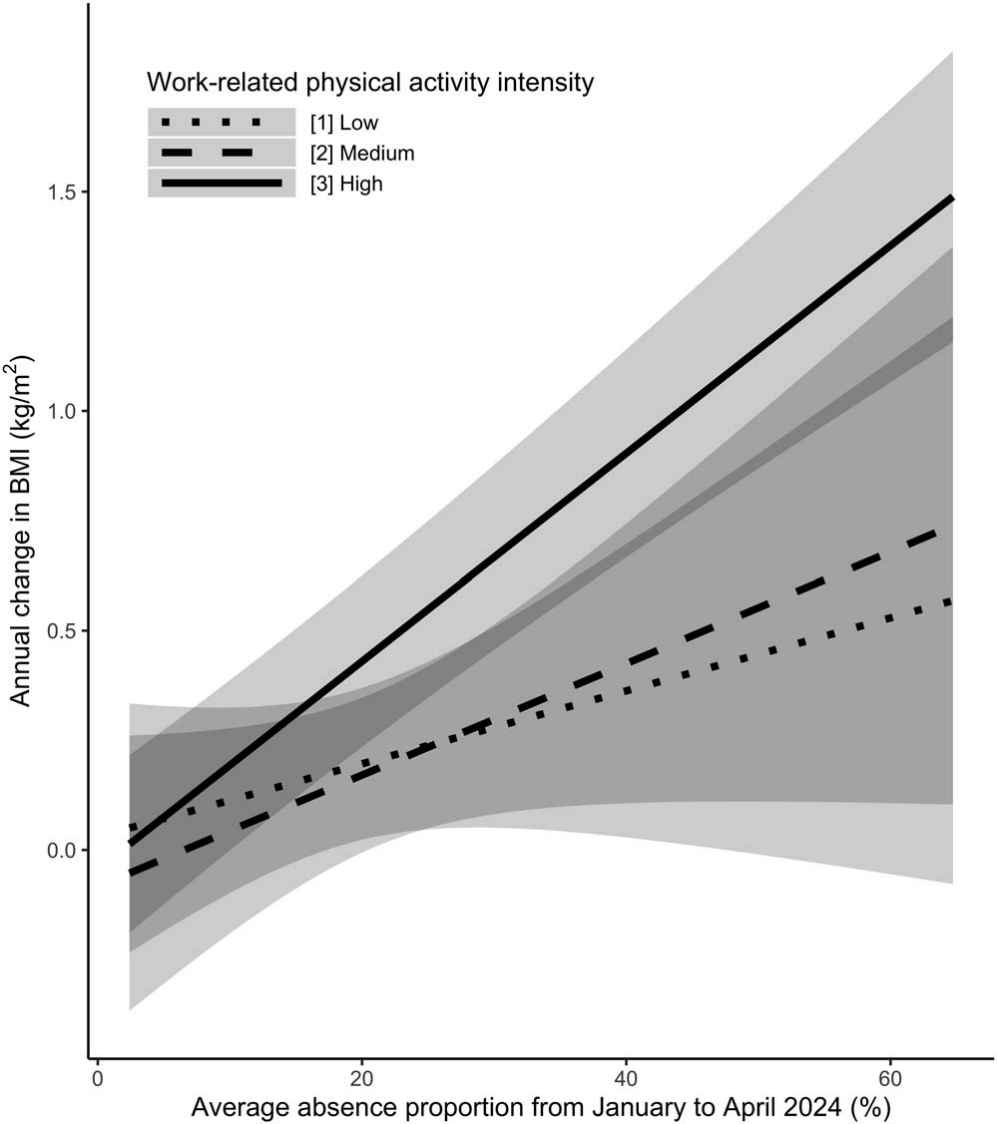
Association between the absence proportion and annual BMI change. The lines illustrate the estimated association between the absence proportion among the scheduled working days and annual BMI change for each physical activity intensity on the work. The shaded areas show 95% CIs.

In the multivariable adjusted model, a significant effect modification of absence proportion on work-related physical activity intensity was observed for BMI (*P* for effect modification = .016; see Figure 3 and Table 3). Specifically, a higher absence proportion was significantly associated with increased BMI in the high-intensity work group (annual change per 20% increase of absence proportion: 0.47; 95% CI: 0.37, 0.58; *P* < .001). Although similar positive associations were observed in low- and medium-intensity groups, these were not statistically significant. Furthermore, a significant effect modification was also observed for SBP (*P* for effect modification <.001). A higher absence proportion significantly increased SBP in medium-intensity (annual change per 20% increase of absence proportion: 2.55; 95% CI: 0.79, 4.31; *P* = .005) and high-intensity groups (annual change per 20% increase of absence proportion: 1.13; 95% CI: 0.13, 2.13; *P* = .027), but not in the low-intensity group. In contrast, there were no significant effect modifications or main effects for DBP, HbA1c, TG, HDL cholesterol, LDL cholesterol, or uric acid levels across different work intensity categories. Although some trends were noted, none reached statistical significance. These patterns were broadly consistent with the results from the unadjusted models, in which the estimates did not materially differ from those of the multivariable analyses.

**Table 3:** The association between the absence proportion and the annual outcome changes among the subpopulation who underwent health checkups in April 2024.

		**Unadjusted model** ^**a**^		**Multivariable adjusted model** ^**b**^	
**Outcome**	**Work intensity**	**Annual outcome change per 20% absence proportion [95% CI] (*P* value)**	***P* value for effect modification**	**Annual outcome change per 20% absence proportion [95% CI] (*P* value)**	***P* value for effect modification**
	Low	0.18 [−0.08 to 0.43] (.171)		0.17 [−0.11 to 0.44] (.230)	.016
BMI	Medium	0.24 [0.05 to 0.43] (.012)	.033	0.25 [−0.03 to 0.53] (.074)	
	High	0.45 [0.33 to 0.58] (<.001)		0.47 [0.37 to 0.58] (<.001)	
	Low	−1.27 [−2.74 to 0.20] (.090)		−0.84 [−1.91 to 0.23] (.124)	<.001
SBP	Medium	2.98 [0.58 to 5.37] (.015)	<.001	2.55 [0.79 to 4.31] (.005)	
	High	1.44 [0.26 to 2.61] (.016)		1.13 [0.13 to 2.13] (.027)	
	Low	−0.10 [−2.91 to 2.71] (.945)		0.04 [−2.71 to 2.79] (.978)	.751
DBP	Medium	2.06 [−0.15 to 4.26] (.067)	.133	1.13 [−1.13 to 3.39] (.327)	
	High	0.80 [−0.17 to 1.77] (.104)		0.59 [−0.50 to 1.67] (0290)	
	Low	−0.03 [−0.07 to 0.02] (.254)		−0.02 [−0.08 to 0.03] (.391)	.219
HbA1c	Medium	0.01 [−0.05 to 0.07] (.715)	.5	0.02 [−0.02 to 0.07] (.300)	
	High	0.00 [−0.02 to 0.02] (.891)		−0.00 [−0.02 to 0.02] (.918)	
	Low	−13.69 [−53.23 to 25.85] (.497)		−23.90 [−63.66 to 15.87] (.238)	.329
TG	Medium	−1.19 [−18.91 to 16.54] (.896)	.466	7.90 [−4.07 to 19.87] (.195)	
	High	8.06 [−2.48 to 18.60] (.133)		5.23 [−4.79 to 15.25] (.306)	
	Low	0.42 [−2.29 to 3.13] (.762)		1.09 [−1.11 to 3.29] (.330)	.054
HDL	Medium	−3.04 [−6.65 to 0.57] (.098)	.237	−3.10 [−6.63 to 0.43] (.085)	
	High	−1.01 [−2.55 to 0.53] (.197)		−1.05 [−2.57 to 0.46] (.172)	
	Low	3.58 [−3.83 to 10.99] (.343)		4.93 [−2.66 to 12.52] (.202)	.606
LDL	Medium	0.56 [−4.69 to 5.80] (.835)	.684	0.73 [−4.02 to 5.48] (.764)	
	High	0.07 [−2.82 to 2.97] (.960)		1.10 [−1.42 to 3.62] (.393)	
	Low	0.20 [−0.03 to 0.44] (.088)		0.19 [−0.05 to 0.42] (.114)	.311
Uric acid	Medium	−0.04 [−0.19 to 0.11] (.578)	.228	−0.01 [−0.16 to 0.13] (.846)	
	High	0.02 [−0.13 to 0.17] (.798)		0.02 [−0.13 to 0.18] (.759)	

Abbreviations: BMI, body mass index; DBP, diastolic blood pressure; HbA1c, hemoglobin A1c; HDL, high-density lipoprotein; LDL, low-density lipoprotein; SBP, systolic blood pressure.

^a^The estimated annual changes show how much the annual outcome varies when the absence proportion increases by 20%. The *P* value for effect modification indicates whether the association of the absence proportions varies depending on the physical activity level on the job.

^b^In the multivariable adjusted models, participants’ age, sex, drinking status, smoking status, exercise habits, commute method, and health metrics measured at the 2023 health examination (HbA1c, triglycerides, HDL cholesterol, LDL cholesterol, SBP, DBP, and uric acid levels) were considered as covariates.

## 4. Discussion

The findings from this study suggest that an abrupt workplace disruption—specifically, a forced shipment suspension that required employees to stay at home—had heterogeneous effects on employees’ cardiometabolic outcomes. Notably, employees engaged in medium-intensity and high-intensity occupations experienced discernible increases in BMI, with the high-intensity group particularly susceptible to weight gain as the absence proportion increased. These results are consistent with evidence that sudden changes in occupational routines can influence daily energy balance, thereby modifying body weight and metabolic markers.^1,2^ The reliance of physically demanding workers on the workplace environment for a substantial portion of their physical activity may explain why those in higher-intensity roles demonstrated the most pronounced BMI escalation. Furthermore, these findings also suggest a dose–response relationship with the duration of stay-at-home periods, indicating that employees with greater baseline physical activity levels may experience larger increases in BMI and related health outcomes as the duration of staying at home extends.

Comparison with studies conducted during the COVID-19 era reinforces these observations, insofar as pandemic-related lockdowns and remote work arrangements were often linked to reduced energy expenditure and weight gain in certain populations.^10,11^ However, remote work offered opportunities for self-directed exercise and healthier home-cooking habits among some employees, particularly in white-collar roles suited to at-home tasks. By contrast, the employees in physically demanding positions at the automobile manufacturer studied here were less able to replicate job-related energy expenditure while absent from on-site work. A significant body of research indicates that job strain and the abrupt loss of routine can undermine health behaviors and amplify stress, further contributing to adverse outcomes.^12^

The significant effect modification of the absence proportion on the occupational intensity for SBP is equally notable. A higher rate of missed workdays was associated with increased SBP among those in medium- and high-intensity roles, but not in low-intensity roles. This finding implies that in addition to potential weight gain, psychosocial stress or disrupted sleep patterns may have contributed to acute blood pressure elevations, particularly among workers whose physical tasks cannot be easily transferred to a home setting. Although DBP and metabolic indices like HbA1c and lipids did not show significant changes, it remains plausible that a longer duration of forced disruption would be required for such markers to reach clinically meaningful thresholds.^13^

Our study has several limitations. First, our study population consisted of the employees of a single company and the majority of them were male. In addition, we excluded individuals with severe preexisting conditions. These limit the generalizability of the findings. Second, unmeasured external factors could have coincided with the suspension and influenced outcomes, despite the abrupt nature of the event. Third, although work-related physical activity intensity classifications were defined based on questionnaires, misclassification may have occurred if certain roles combined unexpected sedentary tasks or if employees changed jobs within the observed years. In future work, it will be necessary to use clear criteria for intensity levels of work—such as those defined by the WHO—before collecting data.^14^ Finally, because different individuals contributed data in different months, residual seasonality cannot be fully excluded. Although seasonal fixed effects could, in principle, be added, the study spanned fewer than 2 complete annual cycles, which would render such adjustments weakly identified and potentially over-fitted. As a pragmatic alternative, we provide month-by-month descriptive checks for 2023 (Table S1), which suggest only modest between-month differences in most characteristics.

Despite these limitations, the results underscore the need for targeted interventions when workplace disruptions arise. Organizations might consider providing structured at-home exercise programs or virtual coaching sessions to employees in physically demanding roles, thereby mitigating the abrupt loss of energy expenditure.^15^ Additionally, stress-management resources and social support networks could help workers adapt to the uncertainty of forced absences.^16^ In the context of public health planning, companies that rely on manual labor may benefit from contingency strategies that include rotating shifts or partial on-site operations to maintain a degree of routine occupational activity, thereby reducing the potential health risks associated with prolonged inactivity.^11^

## 5. Conclusions

In summary, this study found that the associations of the exposure of stay-at-home with changes in BMI and cardiometabolic factors differ according to occupational physical activity level. Workers with high occupational activity experienced greater weight gain and adverse metabolic changes during a period of forced absence, compared with those with low occupational activity. These results suggest that maintaining physical activity during periods of work interruption is crucial, particularly for individuals whose jobs are physically active. Public health initiatives should consider tailored recommendations to help active workers remain active when they are unable to attend work. Future research is warranted to explore interventions that can prevent weight gain and metabolic deterioration during such periods, and to examine the long-term health outcomes once normal work resumes.

## Supplementary Material

Web_Material_uiaf069

## Data Availability

The dataset analyzed in this study comprises confidential employee information. Due to privacy concerns and ethical considerations, raw data cannot be made publicly available. However, inquiries regarding the analytic methods, statistical programming code, or further methodological details can be directed to the corresponding author upon reasonable request.
