# Supplementary material for: Different associations of stay-at-home exposure with changes in body mass index and cardiometabolic factors depending on occupational physical activity: a longitudinal quasi-experimental design
Source: J Occup Health. 2025 Nov 28;68(1):uiaf069. doi: 10.1093/joccuh/uiaf069 (PMC12788860; doi:10.1093/joccuh/uiaf069)
Supplement: Web_Material_uiaf069 [file web_material_uiaf069.pdf]

Supplementary Materials

For Review Only

Table S1. Demographic and clinical characteristics by month in 2023

	Work-related physical activity intensity: Low												
	**Overall**	**January**	**February**	**March**	**April**	**May**	**June**	**July**	**August**	**September**	**October**	**November**	**December**
	N = 3,360	N = 284	N = 250	N = 284	N = 249	N = 243	N = 291	N = 323	N = 290	N = 294	N = 310	N = 289	N = 253
Age	39.96 (31.98, 49.99)	42.01 (33.19, 53.02)	40.01 (31.04, 53.00)	38.00 (32.01, 50.52)	38.98 (32.00, 46.97)	39.02 (32.22, 50.03)	40.01 (32.01, 50.01)	40.99 (30.99, 50.04)	40.95 (33.02, 49.02)	39.00 (30.94, 49.00)	39.00 (30.93, 48.05)	39.92 (31.01, 49.03)	40.07 (32.98, 50.02)
(Missing)	0% (0)	0% (0)	0% (0)	0% (0)	0% (0)	0% (0)	0% (0)	0% (0)	0% (0)	0% (0)	0% (0)	0% (0)	0% (0)
Sex (Male)	83% (2,772)	81% (231)	82% (204)	81% (229)	81% (202)	86% (208)	81% (235)	81% (263)	82% (237)	83% (245)	84% (261)	83% (241)	85% (216)
(Missing)	0% (0)	0% (0)	0% (0)	0% (0)	0% (0)	0% (0)	0% (0)	0% (0)	0% (0)	0% (0)	0% (0)	0% (0)	0% (0)
BMI	22.11 (20.29, 24.01)	22.14 (20.31, 24.04)	22.25 (20.31, 23.92)	22.00 (20.11, 23.98)	21.82 (20.27, 24.00)	22.20 (20.41, 24.42)	22.13 (20.08, 24.08)	21.92 (20.06, 23.68)	22.23 (20.22, 24.13)	22.24 (20.01, 24.24)	22.17 (20.53, 23.79)	22.22 (20.38, 24.16)	21.95 (20.40, 23.69)
(Missing)	0.1% (4)	0% (0)	0% (0)	0% (0)	0.4% (1)	0% (0)	0% (0)	0.6% (2)	0% (0)	0% (0)	0% (0)	0.3% (1)	0% (0)
Alcohol													
[1] Non-drinker	23% (775)	25% (70)	23% (58)	21% (60)	22% (54)	22% (54)	19% (56)	23% (75)	27% (78)	24% (72)	21% (66)	24% (70)	25% (62)
[2] Occasional	24% (808)	25% (72)	23% (58)	19% (54)	23% (57)	28% (67)	24% (69)	23% (75)	26% (76)	24% (71)	25% (77)	24% (70)	25% (62)
[3] <3days/week	27% (904)	27% (76)	30% (74)	33% (93)	33% (82)	28% (68)	27% (78)	24% (77)	20% (57)	24% (71)	26% (82)	29% (85)	24% (61)

For Review Only

[4] >=3days/week	14% (460)	14% (40)	11% (28)	15% (44)	14% (34)	13% (31)	16% (47)	15% (49)	14% (40)	12% (36)	13% (41)	11% (31)	15% (39)
[5] Everyday	12% (412)	9.2% (26)	13% (32)	12% (33)	8.8% (22)	9.5% (23)	14% (40)	15% (47)	13% (39)	15% (44)	14% (44)	11% (33)	11% (29)
(Missing)	<0.1% (1)	0% (0)	0% (0)	0% (0)	0% (0)	0% (0)	0.3% (1)	0% (0)	0% (0)	0% (0)	0% (0)	0% (0)	0% (0)
Smoking													
[1] Never	65% (2,180)	60% (171)	70% (174)	65% (184)	66% (165)	67% (163)	66% (193)	64% (208)	62% (179)	64% (187)	65% (203)	65% (189)	65% (164)
[2] Past	18% (617)	20% (57)	17% (43)	18% (50)	18% (45)	18% (44)	16% (47)	17% (56)	21% (62)	20% (58)	17% (54)	20% (58)	17% (43)
[3] Current	17% (562)	20% (56)	13% (33)	18% (50)	16% (39)	15% (36)	18% (51)	18% (59)	17% (49)	17% (49)	17% (53)	15% (42)	18% (45)
(Missing)	<0.1% (1)	0% (0)	0% (0)	0% (0)	0% (0)	0% (0)	0% (0)	0% (0)	0% (0)	0% (0)	0% (0)	0% (0)	0.4% (1)
Exercise (Yes)	31% (1,030)	25% (71)	33% (83)	28% (80)	29% (71)	35% (84)	28% (82)	33% (107)	33% (95)	33% (96)	28% (87)	30% (86)	35% (88)
(Missing)	0.1% (4)	0.4% (1)	0.4% (1)	0% (0)	0% (0)	0% (0)	0.3% (1)	0% (0)	0% (0)	0.3% (1)	0% (0)	0% (0)	0% (0)
Commute													
Bicycle	14% (482)	14% (41)	15% (38)	14% (41)	14% (35)	13% (32)	16% (45)	12% (40)	12% (34)	15% (43)	17% (54)	14% (41)	15% (38)
Car/Motorbike	41% (1,366)	38% (108)	41% (103)	45% (127)	39% (96)	36% (88)	40% (117)	46% (148)	41% (118)	41% (121)	37% (114)	44% (128)	39% (98)
Train/Bus	30% (1,006)	33% (92)	28% (69)	25% (70)	35% (88)	33% (81)	33% (95)	26% (85)	32% (92)	26% (76)	31% (96)	30% (86)	30% (76)
Walking	15% (500)	15% (42)	16% (40)	16% (45)	12% (29)	17% (41)	11% (33)	15% (50)	16% (45)	18% (54)	15% (46)	12% (34)	16% (41)
(Missing)	0.2% (6)	0.4% (1)	0% (0)	0.4% (1)	0.4% (1)	0.4% (1)	0.3% (1)	0% (0)	0.3% (1)	0% (0)	0% (0)	0% (0)	0% (0)
Location													

For Review Only

Osaka 1	79% (2,669)	79% (224)	78% (194)	78% (221)	81% (202)	81% (196)	80% (233)	75% (241)	82% (239)	82% (242)	78% (243)	79% (228)	81% (206)
Osaka 2	9.3% (311)	5.3% (15)	8.8% (22)	9.5% (27)	11% (27)	11% (27)	8.9% (26)	11% (37)	7.9% (23)	7.1% (21)	11% (34)	9.7% (28)	9.5% (24)
Kyoto	1.5% (52)	2.8% (8)	2.8% (7)	2.1% (6)	0.4% (1)	0.4% (1)	1.7% (5)	1.2% (4)	1.4% (4)	2.0% (6)	0.6% (2)	2.4% (7)	0.4% (1)
Shiga 1	5.0% (167)	5.6% (16)	5.2% (13)	6.0% (17)	3.6% (9)	3.7% (9)	3.8% (11)	6.8% (22)	4.5% (13)	3.4% (10)	6.1% (19)	5.2% (15)	5.1% (13)
Shiga 2	4.8% (161)	7.4% (21)	5.6% (14)	4.6% (13)	4.0% (10)	4.1% (10)	5.5% (16)	5.9% (19)	3.8% (11)	5.1% (15)	3.9% (12)	3.8% (11)	3.6% (9)
(Missing)	0% (0)	0% (0)	0% (0)	0% (0)	0% (0)	0% (0)	0% (0)	0% (0)	0% (0)	0% (0)	0% (0)	0% (0)	0% (0)
HbA1c	5.50 (5.30, 5.70)	5.60 (5.30, 5.70)	5.50 (5.30, 5.70)	5.50 (5.30, 5.70)	5.50 (5.30, 5.60)	5.60 (5.35, 5.70)	5.60 (5.40, 5.70)	5.50 (5.30, 5.70)	5.50 (5.30, 5.60)	5.50 (5.30, 5.70)	5.50 (5.30, 5.70)	5.50 (5.40, 5.70)	5.60 (5.40, 5.70)
(Missing)	25% (838)	21% (61)	28% (70)	25% (71)	25% (62)	28% (67)	24% (70)	25% (82)	21% (60)	30% (89)	24% (75)	26% (74)	23% (57)
Triglycerides	79.00 (57.00, 113.00)	80.00 (57.00, 117.00)	76.00 (52.50, 103.00)	72.50 (57.00, 106.00)	75.00 (55.00, 101.00)	73.50 (56.00, 115.00)	82.00 (54.00, 121.00)	78.00 (57.00, 108.00)	90.50 (61.00, 122.00)	83.00 (59.00, 123.00)	75.00 (57.00, 111.00)	85.00 (60.00, 119.00)	81.00 (57.50, 113.00)
(Missing)	25% (831)	21% (61)	28% (70)	25% (70)	25% (62)	27% (65)	24% (70)	25% (82)	21% (60)	30% (87)	24% (75)	25% (72)	23% (57)
HDL	59.00 (50.00, 70.00)	58.00 (50.00, 73.00)	61.00 (51.00, 75.00)	59.00 (49.00, 71.00)	59.00 (49.00, 69.00)	60.50 (51.00, 73.00)	59.00 (49.00, 68.00)	59.00 (49.00, 68.00)	58.00 (49.00, 69.00)	60.00 (51.00, 69.00)	59.00 (50.00, 69.00)	59.00 (51.00, 69.00)	61.00 (51.00, 74.00)
(Missing)	25% (831)	21% (61)	28% (70)	25% (70)	25% (62)	27% (65)	24% (70)	25% (82)	21% (60)	30% (87)	24% (75)	25% (72)	23% (57)

For Review Only

LDL	119.00 (101.00, 139.00)	125.00 (105.00, 145.00)	121.50 (101.50, 139.00)	118.50 (102.00, 135.00)	123.00 (104.00, 139.00)	117.50 (97.00, 139.00)	114.00 (97.00, 134.00)	117.00 (102.00, 132.00)	119.00 (100.00, 139.00)	118.00 (96.00, 139.00)	118.00 (100.00, 139.00)	128.00 (106.00, 147.00)	120.00 (102.00, 143.00)
(Missing)	25% (831)	21% (61)	28% (70)	25% (70)	25% (62)	27% (65)	24% (70)	25% (82)	21% (60)	30% (87)	24% (75)	25% (72)	23% (57)
SBP	116.00 (107.00, 125.00)	117.00 (109.00, 126.00)	116.00 (110.00, 125.00)	114.00 (106.00, 124.00)	116.00 (106.00, 126.00)	117.00 (108.00, 126.00)	113.00 (106.00, 123.00)	116.00 (107.00, 124.00)	117.00 (106.00, 126.00)	116.00 (108.00, 124.00)	116.00 (108.00, 124.00)	115.00 (106.00, 124.00)	116.00 (108.00, 126.00)
(Missing)	0% (0)	0% (0)	0% (0)	0% (0)	0% (0)	0% (0)	0% (0)	0% (0)	0% (0)	0% (0)	0% (0)	0% (0)	0% (0)
DBP	75.00 (68.00, 82.00)	76.50 (69.50, 84.00)	75.00 (68.00, 82.00)	73.00 (66.00, 81.00)	75.00 (69.00, 82.00)	76.00 (68.00, 83.00)	73.00 (67.00, 81.00)	74.00 (67.00, 82.00)	76.00 (68.00, 83.00)	74.00 (68.00, 81.00)	75.50 (69.00, 81.00)	74.00 (67.00, 82.00)	76.00 (68.00, 82.00)
(Missing)	0% (0)	0% (0)	0% (0)	0% (0)	0% (0)	0% (0)	0% (0)	0% (0)	0% (0)	0% (0)	0% (0)	0% (0)	0% (0)
Uric acid	5.90 (5.00, 6.70)	5.80 (5.00, 6.60)	5.50 (4.70, 6.45)	5.80 (4.80, 6.60)	5.80 (5.00, 6.70)	5.95 (5.10, 6.90)	6.10 (5.00, 6.80)	5.90 (4.90, 6.80)	5.90 (5.00, 6.80)	5.90 (4.90, 6.80)	5.80 (5.20, 6.80)	6.00 (5.10, 7.00)	5.80 (4.90, 6.65)
(Missing)	25% (831)	21% (61)	28% (70)	25% (70)	25% (62)	27% (65)	24% (70)	25% (82)	21% (60)	30% (87)	24% (75)	25% (72)	23% (57)
**Work-related physical activity intensity: Medium**													
	**Overall** N = 1,534	**January** N = 113	**February** N = 128	**March** N = 124	**April** N = 130	**May** N = 118	**June** N = 131	**July** N = 134	**August** N = 137	**September** N = 148	**October** N = 129	**November** N = 136	**December** N = 106
Age	39.02 (33.96, 47.95)	37.98 (31.98, 49.03)	40.95 (35.05, 51.00)	39.02 (34.04, 46.97)	37.04 (32.04, 46.05)	39.02 (34.98, 49.96)	37.98 (33.95, 44.02)	40.97 (33.03, 50.10)	39.03 (33.01, 45.96)	40.02 (33.44, 48.02)	39.01 (33.07, 46.00)	40.99 (35.97, 48.54)	38.00 (33.03, 46.00)
(Missing)	0% (0)	0% (0)	0% (0)	0% (0)	0% (0)	0% (0)	0% (0)	0% (0)	0% (0)	0% (0)	0% (0)	0% (0)	0% (0)

Sex (Male)	94% (1,448)	95% (107)	96% (123)	97% (120)	92% (119)	92% (109)	95% (124)	93% (124)	95% (130)	95% (140)	92% (119)	97% (132)	95% (101)
(Missing)	0% (0)	0% (0)	0% (0)	0% (0)	0% (0)	0% (0)	0% (0)	0% (0)	0% (0)	0% (0)	0% (0)	0% (0)	0% (0)
BMI	22.37 (20.67, 24.37)	22.81 (20.69, 24.58)	22.34 (20.90, 24.42)	22.49 (20.77, 23.94)	22.56 (21.27, 24.24)	22.84 (20.78, 24.66)	22.21 (20.56, 24.76)	22.01 (20.09, 24.71)	22.17 (20.55, 23.75)	22.21 (20.99, 24.48)	22.22 (20.47, 23.98)	22.79 (20.54, 24.51)	22.33 (20.30, 24.56)
(Missing)	<0.1% (1)	0% (0)	0% (0)	0% (0)	0% (0)	0% (0)	0% (0)	0% (0)	0.7% (1)	0% (0)	0% (0)	0% (0)	0% (0)
Alcohol													
[1] Non-drinker	23% (350)	23% (26)	30% (39)	23% (29)	25% (32)	18% (21)	20% (26)	20% (27)	19% (26)	22% (33)	25% (32)	25% (34)	24% (25)
[2] Occasional	22% (330)	21% (24)	21% (27)	22% (27)	21% (27)	22% (26)	24% (31)	17% (23)	23% (31)	22% (32)	24% (31)	20% (27)	23% (24)
[3] <3days/week	23% (345)	23% (26)	13% (16)	19% (24)	31% (40)	27% (32)	21% (27)	30% (40)	27% (37)	19% (28)	20% (26)	18% (24)	24% (25)
[4] >=3days/week	16% (241)	15% (17)	19% (24)	17% (21)	15% (20)	15% (18)	15% (20)	18% (24)	7.3% (10)	21% (31)	12% (15)	18% (24)	16% (17)
[5] Everyday	17% (267)	18% (20)	17% (22)	19% (23)	8.5% (11)	18% (21)	20% (26)	15% (20)	24% (33)	16% (24)	19% (25)	20% (27)	14% (15)
(Missing)	<0.1% (1)	0% (0)	0% (0)	0% (0)	0% (0)	0% (0)	0.8% (1)	0% (0)	0% (0)	0% (0)	0% (0)	0% (0)	0% (0)
Smoking													
[1] Never	46% (704)	44% (50)	42% (54)	38% (47)	50% (64)	48% (57)	40% (51)	49% (66)	47% (65)	45% (66)	50% (64)	50% (68)	49% (52)
[2] Past	20% (311)	24% (27)	25% (32)	20% (25)	21% (27)	21% (25)	23% (30)	18% (24)	15% (20)	23% (34)	15% (19)	19% (26)	21% (22)
[3] Current	34% (516)	32% (36)	33% (42)	42% (52)	29% (38)	31% (36)	37% (48)	33% (44)	38% (52)	32% (48)	36% (46)	31% (42)	30% (32)
(Missing)	0.2% (3)	0% (0)	0% (0)	0% (0)	0.8% (1)	0% (0)	1.5% (2)	0% (0)	0% (0)	0% (0)	0% (0)	0% (0)	0% (0)
Exercise (Yes)	29% (443)	26% (29)	28% (36)	27% (33)	30% (39)	36% (42)	34% (44)	24% (32)	31% (43)	27% (40)	28% (36)	28% (38)	29% (31)

For Review Only

(Missing)	0.2% (3)	0% (0)	0% (0)	0% (0)	0.8% (1)	0% (0)	0% (0)	0.7% (1)	0% (0)	0% (0)	0.8% (1)	0% (0)	0% (0)
Commute													
Bicycle	12% (186)	9.7% (11)	10% (13)	8.9% (11)	11% (14)	9.3% (11)	9.9% (13)	14% (19)	15% (21)	14% (21)	12% (15)	18% (24)	12% (13)
Car/Motorbike	58% (891)	62% (70)	65% (83)	65% (80)	60% (78)	54% (64)	55% (72)	59% (79)	54% (74)	55% (82)	53% (69)	58% (79)	58% (61)
Train/Bus	17% (263)	17% (19)	15% (19)	15% (18)	18% (23)	21% (25)	18% (24)	18% (24)	15% (20)	22% (32)	18% (23)	15% (20)	15% (16)
Walking	13% (194)	12% (13)	10% (13)	12% (15)	12% (15)	15% (18)	17% (22)	9.0% (12)	16% (22)	8.8% (13)	17% (22)	9.6% (13)	15% (16)
(Missing)	0% (0)	0% (0)	0% (0)	0% (0)	0% (0)	0% (0)	0% (0)	0% (0)	0% (0)	0% (0)	0% (0)	0% (0)	0% (0)
Location													
Osaka 1	49% (745)	42% (47)	48% (61)	48% (59)	50% (65)	47% (55)	43% (56)	49% (66)	53% (72)	51% (76)	55% (71)	47% (64)	50% (53)
Osaka 2	3.2% (49)	4.4% (5)	2.3% (3)	1.6% (2)	3.1% (4)	4.2% (5)	3.8% (5)	3.0% (4)	1.5% (2)	2.0% (3)	3.9% (5)	4.4% (6)	4.7% (5)
Kyoto	8.7% (133)	11% (12)	11% (14)	10% (13)	6.9% (9)	11% (13)	7.6% (10)	6.0% (8)	5.1% (7)	13% (19)	7.8% (10)	6.6% (9)	8.5% (9)
Shiga 1	22% (342)	29% (33)	23% (29)	26% (32)	29% (38)	19% (22)	23% (30)	19% (26)	20% (27)	18% (27)	18% (23)	24% (32)	22% (23)
Shiga 2	17% (265)	14% (16)	16% (21)	15% (18)	11% (14)	19% (23)	23% (30)	22% (30)	21% (29)	16% (23)	16% (20)	18% (25)	15% (16)
(Missing)	0% (0)	0% (0)	0% (0)	0% (0)	0% (0)	0% (0)	0% (0)	0% (0)	0% (0)	0% (0)	0% (0)	0% (0)	0% (0)
HbA1c	5.50 (5.30, 5.70)	5.60 (5.40, 5.80)	5.50 (5.30, 5.70)	5.50 (5.40, 5.70)	5.50 (5.40, 5.70)	5.50 (5.40, 5.70)	5.50 (5.30, 5.70)	5.60 (5.30, 5.80)	5.50 (5.30, 5.60)	5.55 (5.40, 5.70)	5.50 (5.30, 5.70)	5.50 (5.30, 5.70)	5.60 (5.40, 5.80)
(Missing)	25% (384)	33% (37)	16% (21)	23% (28)	25% (33)	19% (23)	25% (33)	23% (31)	30% (41)	27% (40)	30% (39)	17% (23)	33% (35)
Triglycerides	79.00 (56.00, 114.00)	78.50 (54.00, 103.00)	78.00 (52.00, 114.00)	81.00 (60.00, 115.00)	77.00 (50.00, 115.00)	74.00 (54.00, 115.00)	80.00 (57.00, 112.00)	66.00 (48.00, 118.00)	79.50 (56.00, 104.00)	75.00 (55.50, 107.50)	82.00 (63.00, 121.00)	83.00 (64.00, 115.00)	82.00 (59.00, 126.00)
(Missing)	25% (383)	33% (37)	16% (21)	23% (28)	25% (33)	19% (22)	25% (33)	23% (31)	30% (41)	27% (40)	30% (39)	17% (23)	33% (35)

For Review Only

HDL	58.00 (50.00, 68.00)	60.50 (53.00, 69.50)	59.00 (50.00, 69.00)	59.00 (49.50, 69.00)	56.00 (50.00, 63.00)	58.00 (49.00, 69.50)	56.00 (49.00, 71.00)	61.00 (51.00, 74.00)	55.00 (50.00, 67.00)	60.00 (47.50, 66.00)	57.50 (50.00, 67.00)	58.00 (52.00, 67.00)	60.00 (54.00, 69.00)
(Missing)	25% (383)	33% (37)	16% (21)	23% (28)	25% (33)	19% (22)	25% (33)	23% (31)	30% (41)	27% (40)	30% (39)	17% (23)	33% (35)
LDL	117.00 (100.00, 137.00)	123.50 (103.00, 151.50)	117.00 (102.00, 137.00)	115.00 (101.50, 142.00)	125.00 (106.00, 145.00)	119.50 (104.00, 141.00)	112.50 (89.00, 133.00)	113.00 (92.00, 126.00)	113.50 (100.00, 129.50)	112.50 (97.00, 131.50)	113.00 (103.00, 129.00)	122.00 (106.00, 139.00)	124.00 (100.00, 140.00)
(Missing)	25% (383)	33% (37)	16% (21)	23% (28)	25% (33)	19% (22)	25% (33)	23% (31)	30% (41)	27% (40)	30% (39)	17% (23)	33% (35)
SBP	118.00 (109.00, 126.00)	118.00 (112.00, 126.00)	120.00 (114.00, 127.00)	118.00 (110.00, 127.00)	120.00 (111.00, 128.00)	118.50 (108.00, 126.00)	116.00 (108.00, 124.00)	116.00 (107.00, 123.00)	117.00 (107.00, 125.00)	117.00 (110.50, 125.00)	117.00 (108.00, 125.00)	118.00 (108.00, 127.50)	120.50 (112.00, 128.00)
(Missing)	0% (0)	0% (0)	0% (0)	0% (0)	0% (0)	0% (0)	0% (0)	0% (0)	0% (0)	0% (0)	0% (0)	0% (0)	0% (0)
DBP	76.00 (69.00, 82.00)	78.00 (71.00, 83.00)	78.00 (71.00, 83.00)	75.00 (69.00, 82.00)	76.50 (70.00, 83.00)	74.00 (68.00, 82.00)	74.00 (67.00, 81.00)	75.00 (68.00, 82.00)	76.00 (67.00, 82.00)	75.00 (69.00, 81.00)	74.00 (68.00, 81.00)	77.00 (71.00, 83.00)	77.50 (71.00, 84.00)
(Missing)	0% (0)	0% (0)	0% (0)	0% (0)	0% (0)	0% (0)	0% (0)	0% (0)	0% (0)	0% (0)	0% (0)	0% (0)	0% (0)
Uric acid	6.00 (5.20, 6.80)	5.90 (5.40, 7.15)	5.90 (5.10, 6.70)	6.20 (5.15, 7.05)	5.90 (5.20, 6.70)	6.00 (5.15, 6.95)	6.20 (5.40, 7.10)	6.10 (5.10, 6.90)	6.15 (5.30, 6.80)	6.10 (5.40, 6.85)	5.90 (5.10, 6.70)	6.20 (5.30, 6.80)	5.90 (5.00, 6.90)
(Missing)	25% (383)	33% (37)	16% (21)	23% (28)	25% (33)	19% (22)	25% (33)	23% (31)	30% (41)	27% (40)	30% (39)	17% (23)	33% (35)

	**Work-related physical activity intensity: High**												
	**Overall**	**January**	**February**	**March**	**April**	**May**	**June**	**July**	**August**	**September**	**October**	**November**	**December**
	N = 3,413	N = 309	N = 279	N = 323	N = 270	N = 267	N = 295	N = 276	N = 302	N = 252	N = 320	N = 245	N = 275

For Review Only

Age	36.03 (28.01, 43.99)	36.02 (30.00, 41.98)	36.96 (30.96, 44.05)	36.01 (27.97, 44.92)	36.00 (31.00, 43.02)	36.04 (28.09, 44.00)	36.11 (27.06, 44.98)	35.97 (26.01, 43.00)	36.97 (28.01, 44.01)	37.00 (28.98, 44.02)	36.01 (27.01, 43.99)	35.01 (25.97, 44.04)	36.96 (30.99, 44.02)
(Missing)	0% (0)	0% (0)	0% (0)	0% (0)	0% (0)	0% (0)	0% (0)	0% (0)	0% (0)	0% (0)	0% (0)	0% (0)	0% (0)
Sex (Male)	97% (3,300)	97% (299)	97% (272)	97% (314)	97% (261)	96% (255)	95% (281)	97% (269)	96% (291)	97% (244)	96% (306)	97% (237)	99% (271)
(Missing)	0% (0)	0% (0)	0% (0)	0% (0)	0% (0)	0% (0)	0% (0)	0% (0)	0% (0)	0% (0)	0% (0)	0% (0)	0% (0)
BMI	21.73 (19.99, 23.87)	21.88 (20.08, 23.87)	21.73 (20.08, 23.77)	21.49 (19.87, 23.49)	21.82 (20.01, 23.96)	21.78 (20.14, 23.76)	22.00 (19.98, 24.01)	22.00 (19.93, 23.83)	21.34 (19.79, 23.71)	21.55 (19.72, 23.70)	21.92 (20.43, 24.03)	21.87 (20.09, 24.36)	21.66 (19.97, 23.95)
(Missing)	0.2% (7)	0.6% (2)	0.4% (1)	0% (0)	0% (0)	0% (0)	0.3% (1)	0% (0)	0.3% (1)	0.4% (1)	0% (0)	0% (0)	0.4% (1)
Alcohol													
[1] Non-drinker	33% (1,124)	37% (114)	36% (100)	37% (121)	30% (82)	32% (85)	35% (101)	30% (83)	33% (99)	30% (76)	31% (100)	29% (70)	34% (93)
[2] Occasional	24% (800)	23% (71)	22% (61)	25% (80)	27% (72)	23% (61)	26% (77)	26% (71)	21% (64)	21% (54)	25% (79)	25% (61)	18% (49)
[3] <3days/week	18% (608)	20% (61)	18% (51)	15% (49)	19% (50)	17% (46)	14% (40)	18% (50)	21% (62)	19% (48)	18% (58)	17% (42)	19% (51)
[4] >=3days/week	12% (396)	7.8% (24)	9.0% (25)	8.0% (26)	13% (34)	12% (32)	13% (38)	18% (50)	10% (30)	15% (38)	11% (35)	13% (31)	12% (33)
[5] Everyday	14% (471)	12% (38)	14% (40)	15% (47)	12% (32)	15% (41)	12% (36)	8.0% (22)	15% (45)	14% (36)	14% (46)	16% (39)	18% (49)
(Missing)	0.4% (14)	0.3% (1)	0.7% (2)	0% (0)	0% (0)	0.7% (2)	1.0% (3)	0% (0)	0.7% (2)	0% (0)	0.6% (2)	0.8% (2)	0% (0)
Smoking													

For Review Only

[1] Never	44% (1,496)	42% (130)	39% (110)	40% (129)	49% (132)	46% (123)	47% (140)	43% (120)	45% (136)	43% (109)	46% (147)	44% (107)	41% (113)
[2] Past	16% (547)	16% (48)	21% (58)	17% (54)	14% (38)	15% (39)	14% (42)	18% (49)	14% (43)	14% (35)	16% (51)	15% (36)	20% (54)
[3] Current	40% (1,368)	42% (131)	40% (111)	43% (140)	37% (100)	39% (104)	38% (113)	39% (107)	41% (123)	43% (107)	38% (122)	42% (102)	39% (108)
(Missing)	<0.1% (2)	0% (0)	0% (0)	0% (0)	0% (0)	0.4% (1)	0% (0)	0% (0)	0% (0)	0.4% (1)	0% (0)	0% (0)	0% (0)
Exercise (Yes)	32% (1,095)	34% (104)	28% (78)	28% (90)	28% (76)	29% (78)	27% (81)	39% (109)	40% (120)	35% (87)	34% (108)	35% (85)	29% (79)
(Missing)	<0.1% (1)	0% (0)	0% (0)	0% (0)	0% (0)	0% (0)	0% (0)	0% (0)	0% (0)	0% (0)	0.3% (1)	0% (0)	0% (0)
Commute													
Bicycle	7.0% (238)	8.1% (25)	8.2% (23)	7.8% (25)	7.8% (21)	4.1% (11)	8.5% (25)	9.1% (25)	5.6% (17)	6.7% (17)	7.5% (24)	4.5% (11)	5.1% (14)
Car/Motorbike	62% (2,126)	65% (201)	63% (176)	64% (206)	67% (181)	59% (158)	57% (168)	57% (156)	63% (191)	65% (165)	64% (204)	60% (146)	63% (174)
Train/Bus	14% (468)	12% (36)	14% (40)	11% (37)	9.6% (26)	17% (45)	17% (50)	17% (47)	13% (39)	13% (32)	12% (38)	16% (38)	15% (40)
Walking	17% (580)	15% (47)	14% (40)	17% (54)	16% (42)	20% (53)	18% (52)	17% (48)	18% (55)	15% (38)	17% (54)	20% (50)	17% (47)
(Missing)	<0.1% (1)	0% (0)	0% (0)	0.3% (1)	0% (0)	0% (0)	0% (0)	0% (0)	0% (0)	0% (0)	0% (0)	0% (0)	0% (0)
Location													
Osaka 1	12% (403)	13% (41)	12% (34)	11% (34)	11% (30)	13% (35)	15% (43)	11% (29)	13% (40)	12% (29)	11% (34)	12% (30)	8.7% (24)
Osaka 2	2.6% (88)	3.2% (10)	3.9% (11)	2.8% (9)	1.5% (4)	3.4% (9)	1.7% (5)	2.2% (6)	1.7% (5)	3.6% (9)	2.8% (9)	2.9% (7)	1.5% (4)
Kyoto	18% (626)	16% (50)	19% (54)	20% (63)	17% (47)	17% (46)	20% (60)	19% (52)	20% (59)	17% (43)	19% (62)	17% (42)	17% (48)
Shiga 1	33% (1,134)	32% (100)	30% (83)	33% (106)	41% (110)	33% (87)	29% (87)	36% (99)	31% (94)	33% (83)	34% (108)	34% (83)	34% (94)

For Review Only

Shiga 2	34% (1,162)	35% (108)	35% (97)	34% (111)	29% (79)	34% (90)	34% (100)	33% (90)	34% (104)	35% (88)	33% (107)	34% (83)	38% (105)
(Missing)	0% (0)	0% (0)	0% (0)	0% (0)	0% (0)	0% (0)	0% (0)	0% (0)	0% (0)	0% (0)	0% (0)	0% (0)	0% (0)
HbA1c	5.60 (5.40, 5.70)	5.60 (5.40, 5.80)	5.60 (5.40, 5.80)	5.60 (5.40, 5.70)	5.60 (5.40, 5.70)	5.60 (5.40, 5.80)	5.60 (5.40, 5.75)	5.50 (5.30, 5.80)	5.50 (5.30, 5.70)	5.50 (5.30, 5.70)	5.60 (5.40, 5.70)	5.50 (5.40, 5.70)	5.60 (5.40, 5.70)
(Missing)	36% (1,236)	38% (116)	36% (101)	39% (125)	35% (95)	33% (87)	38% (111)	37% (103)	37% (112)	33% (83)	37% (117)	42% (103)	30% (83)
Triglycerides	67.00 (47.00, 100.00)	62.00 (46.00, 95.00)	61.00 (45.00, 86.00)	59.00 (43.00, 84.00)	65.00 (45.00, 97.00)	73.00 (50.00, 100.50)	74.00 (50.00, 112.00)	74.00 (48.00, 119.00)	70.00 (48.00, 108.00)	63.00 (46.00, 98.00)	68.00 (49.00, 99.00)	67.50 (52.00, 93.00)	71.00 (47.00, 107.00)
(Missing)	36% (1,237)	38% (116)	37% (102)	39% (125)	35% (95)	33% (87)	38% (111)	37% (103)	37% (112)	33% (83)	37% (117)	42% (103)	30% (83)
HDL	62.00 (52.00, 72.00)	64.00 (54.00, 74.00)	65.00 (54.00, 74.00)	64.50 (53.00, 75.00)	59.00 (52.00, 68.00)	62.00 (52.00, 72.00)	60.00 (50.50, 69.00)	59.00 (51.00, 69.00)	61.00 (52.00, 70.00)	61.00 (51.00, 70.00)	59.00 (51.00, 71.00)	61.00 (52.00, 73.00)	64.00 (53.00, 79.00)
(Missing)	36% (1,237)	38% (116)	37% (102)	39% (125)	35% (95)	33% (87)	38% (111)	37% (103)	37% (112)	33% (83)	37% (117)	42% (103)	30% (83)
LDL	113.00 (93.00, 133.00)	120.00 (98.00, 142.00)	110.00 (93.00, 134.00)	112.00 (91.00, 131.00)	112.00 (94.00, 132.00)	117.50 (97.00, 137.00)	111.00 (97.00, 131.50)	111.00 (93.00, 128.00)	114.00 (89.00, 130.00)	112.00 (94.00, 126.00)	114.00 (94.00, 132.00)	113.50 (93.00, 133.00)	107.50 (93.00, 130.50)
(Missing)	36% (1,237)	38% (116)	37% (102)	39% (125)	35% (95)	33% (87)	38% (111)	37% (103)	37% (112)	33% (83)	37% (117)	42% (103)	30% (83)

For Review Only

SBP	115.00 (107.00, 123.00)	115.00 (110.00, 123.00)	116.00 (109.00, 125.00)	115.00 (107.00, 124.00)	115.00 (107.00, 124.00)	114.00 (106.00, 123.00)	114.00 (106.00, 122.00)	114.00 (107.00, 122.00)	116.00 (107.00, 123.00)	115.00 (105.00, 123.00)	115.00 (107.00, 123.00)	117.00 (108.00, 126.00)	117.00 (108.00, 124.00)
(Missing)	0% (0)	0% (0)	0% (0)	0% (0)	0% (0)	0% (0)	0% (0)	0% (0)	0% (0)	0% (0)	0% (0)	0% (0)	0% (0)
DBP	72.00 (65.00, 79.00)	72.00 (66.00, 79.00)	74.00 (66.00, 80.00)	73.00 (65.00, 80.00)	73.00 (66.00, 79.00)	72.00 (65.00, 80.00)	71.00 (65.00, 79.00)	71.00 (64.00, 78.00)	72.00 (64.00, 79.00)	72.00 (64.00, 79.00)	72.00 (65.00, 79.00)	73.00 (65.00, 80.00)	73.00 (66.00, 81.00)
(Missing)	0% (0)	0% (0)	0% (0)	0% (0)	0% (0)	0% (0)	0% (0)	0% (0)	0% (0)	0% (0)	0% (0)	0% (0)	0% (0)
Uric acid	6.00 (5.20, 6.90)	6.00 (5.20, 6.80)	5.90 (5.10, 6.60)	5.70 (5.10, 6.50)	6.00 (5.20, 6.70)	6.30 (5.60, 7.00)	6.20 (5.40, 7.10)	6.30 (5.40, 7.30)	6.20 (5.30, 7.10)	5.80 (5.20, 6.70)	6.00 (5.20, 7.00)	6.10 (5.40, 6.80)	5.60 (4.80, 6.40)
(Missing)	36% (1,239)	38% (116)	37% (102)	39% (125)	35% (95)	33% (87)	38% (111)	37% (103)	38% (114)	33% (83)	37% (117)	42% (103)	30% (83)

Abbreviations: BMI, body mass index; SBP, systolic blood pressure; DBP, diastolic blood pressure; HbA1c, hemoglobin A1c; HDL, high density lipoprotein; LDL, low density lipoprotein.

These characteristics were summarized using median (interquartile range; IQR) for continuous variables, and proportion (count) for categorical variables.

For Review Only

Table S2. Absence proportions among scheduled working days

	Work-related physical activity intensity		
**Year-Month**	**[1] Low**	**[2] Medium**	**[3] High**
**2023-1**	6% [0% ,12%]	6% [6% ,12%]	6% [6% ,18%]
**2023-2**	7% [5% ,10%]	10% [5% ,12%]	5% [0% ,10%]
**2023-3**	9% [4% ,11%]	9% [4% ,13%]	9% [4% ,15%]
**2023-4**	10% [5% ,15%]	10% [5% ,15%]	5% [3% ,10%]
**2023-5**	6% [0% ,6%]	6% [6% ,11%]	8% [6% ,14%]
**2023-6**	5% [5% ,9%]	9% [5% ,14%]	20% [11% ,32%]
**2023-7**	7% [5% ,10%]	10% [5% ,14%]	14% [10% ,24%]
**2023-8**	6% [6% ,11%]	11% [6% ,17%]	11% [6% ,17%]
**2023-9**	5% [5% ,10%]	10% [5% ,14%]	5% [5% ,10%]
**2023-10**	7% [5% ,9%]	9% [5% ,14%]	9% [5% ,9%]
**2023-11**	7% [5% ,9%]	9% [5% ,14%]	9% [5% ,11%]
**2023-12**	7% [5% ,12%]	10% [5% ,18%]	15% [10% ,25%]
**2024-1**	6% [3% ,11%]	11% [6% ,22%]	56% [22% ,61%]
**2024-2**	7% [5% ,10%]	10% [5% ,17%]	24% [10% ,52%]
**2024-3**	7% [5% ,12%]	10% [5% ,14%]	14% [7% ,38%]
**2024-4**	10% [5% ,15%]	10% [5% ,18%]	15% [7% ,30%]
**2024-5**	5% [3% ,10%]	5% [5% ,10%]	10% [5% ,15%]
**2024-6**	5% [3% ,10%]	7% [5% ,10%]	10% [5% ,10%]
**2024-7**	7% [4% ,9%]	9% [4% ,11%]	9% [4% ,13%]
**2024-8**	6% [6% ,12%]	12% [6% ,18%]	18% [12% ,24%]
**2024-9**	5% [5% ,10%]	10% [5% ,12%]	7% [5% ,10%]
**2024-10**	5% [2% ,9%]	7% [5% ,9%]	5% [5% ,9%]
**2024-11**	10% [5% ,10%]	10% [5% ,14%]	10% [5% ,19%]

Each cells showed the median proportions and the inter quartile ranges within each Year-Month duration among each population group stratified by the work-related physical activity intensity.

For Review Only
